# Longitudinal associations of meteorological parameters during winter months in Sweden with self-reported symptoms of anxiety in the spring

**DOI:** 10.1007/s00484-025-03098-w

**Published:** 2026-01-19

**Authors:** Auriba Raza, Timo Partonen, Linda L. Magnusson Hanson, Veera Nieminen, Magnus Asp, Hugo Westerlund, Jaana I. Halonen

**Affiliations:** 1 Division of Psychobiology and Epidemiology, Department of Psychology, Stockholm University, Stockholm, SE-106 91 Sweden; 2 School Of Health & Wellbeing, University of Glasgow, Infirmary Wy, G11 6EW Glasgow, Scotland, UK; 3 Department of Healthcare and Social Welfare, Finnish Institute for Health and Welfare, P.O. Box 30, Helsinki, FI-00271 Finland; 4 Department of Public Health, Finnish Institute for Health and Welfare, P.O. Box 30, Helsinki, FI-00271 Finland; 5 School of Educational Sciences and Psychology, University of Eastern Finland, P.O. Box 111, Joensuu, FI-80101 Finland; 6 Department of Community Planning Services, Swedish Meteorological and Hydrological Institute, Norrköping, SE-601 76 Sweden

**Keywords:** Anxiety, Longitudinal study, Rain, Snow, Sunlight, Winter

## Abstract

Anxiety symptoms may be affected by environmental factors. Changes in weather patterns have been linked to various mental health outcomes, but research focusing on wintertime and anxiety is still sparse. Thus, we investigate longitudinal associations between solar radiation, precipitation, and snow days during winter-time and self-reported anxiety symptoms in the following spring. We used data from 14,237 participants of the Swedish Longitudinal Survey of Health who responded to surveys in spring 2016 and 2018. Symptoms of anxiety was assessed using SCL-ANX4, a subscale of the Symptom Checklist-25. Data on the daily solar radiation and precipitation was averaged over November to January and linked to the health data and residence at municipal level for each participant. For snow days, sum of days with snow over the 3-month period was used. Within-individual design using conditional logistic regression was used. Models were adjusted for age, region, and the remaining meteorological variables. Although odds ratios for anxiety in association with 3-month average solar radiation (OR 0.90, 95% CI 0.65–1.24) and precipitation (OR 0.91, 95% CI 0.79–1.05) were on the protective side, these associations were not statistically significant. We neither observed associations between snow days and anxiety symptoms, nor any significant effect modification by age, sex, civil status, job strain, occupational position, region, type of questionnaire, alcohol use, or physical activity (p-values for interactions > 0.05). Our findings do not demonstrate associations between wintertime weather conditions and symptoms of anxiety and call for further research from different geographical areas and populations.

## Introduction

Anxiety is a common symptom marked by persistent feelings of worry, nervousness, or fear that may interfere with an individual’s ability to perform everyday activities. It encompasses numerous conditions, including generalized anxiety disorder (GAD), panic disorder, social anxiety disorder, and specific phobias (Javaid et al. 2023). Anxiety is among the most prevalent mental health issues globally, affecting 301 million individuals in 2019 (Javaid et al. 2023; Vos et al. 2020). According to the Eurobarometer survey conducted in June 2023, nearly half of the European population (46%) had experienced depression or anxiety, within the past 12 months (European Council 2024). The situation is similar in Scandinavia, where mental health surveys indicate that 48–52% of the participants report symptoms of anxiety (If Skadeförsäkring 2025). For instance, in Sweden, the prevalence of self-reported anxiety has increased from 31% in 2011 to 42% in 2021, from which the proportion of severe anxiety was 7% (Public health agency of Sweden 2022).

Environmental factors, including weather, may have substantial effect on mental health. Scientific evidence supports the notion that seasonal changes, variations in sunlight exposure and other weather conditions have been associated with different mental health outcomes (Bulbena et al. 2005; Burns et al. 2021; Cui et al. 2021; Kent et al. 2009; Kim et al. 2021; Komulainen et al. 2022; Liu et al. 2021; Raza et al., 2024; Sarran et al., 2017; Thompson et al. 2018; Thompson et al. 2023; Zhang et al. 2023). While the majority of existing studies on weather have focused on the effects of temperature (Li et al. 2023; Thompson et al. 2018, 2023), research on other meteorological parameters, such as sunlight (Burns et al. 2021; Cui et al. 2021; Kent et al. 2009; Kim et al. 2021; Komulainen et al. 2022; Raza et al., 2024; Sarran et al., 2017), precipitation (Lee et al. 2023), and snow cover, remains limited and findings have been inconsistent (Komulainen et al. 2022; Lee et al. 2023; Sarran et al., 2017; Son & Shin, 2021). Among mental health problems, symptoms of depression (Burns et al. 2021; Cui et al. 2021; Kent et al. 2009; Kim et al. 2021; Komulainen et al. 2022; Lee et al. 2023; Raza et al., 2024; Sarran et al., 2017) have been most extensively studied with respect to weather parameters, while research examining the impact of weather on anxiety is sparse (Bulbena et al. 2005; Zhang et al. 2023). In particular, snow has not previously been investigated in relation to anxiety outcomes, and most existing studies of anxiety are short-term or ecological, underscoring the need for longitudinal population-based analyses such as the present study.

Investigating weather parameters in relation to anxiety is particularly important around the Northern latitude where winters bring prolonged darkness and limited daylight hours. Changing weather conditions due to climate change may make winters even darker and increase the incidence of mental disorders such as winter depression that is already prevalent (Aromaa et al. 2011; Rastad et al., 2005). Climate projections performed by the Swedish Meteorological and Hydrological Institute (SMHI) indicate an increase in winter temperature by 5.8 °C and 4.5 °C in northern and southern Sweden, respectively, by the end of the 21 st century. The corresponding values for precipitation are + 25% for northern and + 11% for southern Sweden (Lind and Kjellström 2008). The duration of snow cover is projected to decline by 40 to 80 days per winter (Eklund et al. 2015). These gradual shifts in weather conditions attributed to climate change are likely to reduce the amount of light due to increased cloud cover, more frequent rainfall, and reduced reflection from snow. Reduced exposure can exacerbate mental health problems for those already affected or increase symptom incidence among healthy individuals This may increase the demand for care and treatment during the winter months. Therefore, research examining the effects of climate-sensitive weather parameters on specific mental health outcomes like anxiety is needed. Our study specifically investigates whether winter weather patterns in Sweden are associated with self-reported anxiety symptoms in the following spring.

Regulation of vitamin D metabolism and circadian rhythms are important biological mechanisms that provide a plausible explanation for the association between sunlight exposure and anxiety. Clinical studies have reported that supplementing with vitamin D, which the skin produces when exposed to sunlight with ultraviolet B radiation in the summer, can help alleviate the severity of anxiety disorders. Vitamin D plays antioxidant, anti-inflammatory, pro-neurogenic, and neuromodulatory roles in the body, which might reduce anxiety (Kim And Jeon 2018; Kouba et al. 2022; Xin et al. 2019). Furthermore, natural sunlight regulates the circadian rhythms, and persistent circadian misalignment or disruption has been linked to various health issues, including anxiety (Coles et al. 2015; Lyall et al. 2018).

In this study, we fill gaps of previous literature by longitudinally examining associations of various weather parameters; solar radiation, precipitation, and snow days during winter months in Sweden on self-reported symptoms of anxiety in the following spring. We use a within individual study design that compares an individual to his/herself at two different time points and thus controls for possible time-invariant confounders, such as genetics, by design.

## Materials and methods

### Study sample

This study was conducted in Sweden (latitudes between the 55th and 69th parallels north) using data from the Swedish Longitudinal Survey of Health (SLOSH), which is designed to be nationally representative of the Swedish working population (Magnusson Hanson et al. 2018). SLOSH was started in 2006 as a biennial nationally representative survey of work-life participation, work environment, and health and well-being. The original sample included all respondents of the 2003 biennial Work Environment Survey (AMU), which itself was drawn from the annual Labour Force Survey (AKU) administered by the Work Environment Authority and Statistics Sweden (Magnusson Hanson et al. 2018; SLOSH 2024). In subsequent years up to 2020, SLOSH was supplemented with respondents from the AMU surveys conducted between 2003 and 2011, and since 2022 additional respondents from the 2013–2019 AMU surveys have also been included (SLOSH 2024). Although attrition has occurred over time, this continuous supplementation has ensured that SLOSH remains broadly representative of the Swedish working population, even in the context of demographic and societal changes since its inception.

Until 2020, two types of questionnaires were sent every two years: one for individuals who were gainfully employed, working an average of ≥ 30% of full-time hours in the three months preceding the survey, and another for those working less than 30% of full-time hours, as well as for the temporarily or permanently unemployed. We included respondents from both types of survey years 2016 and 2018, cumulative cohort size was 40,877 in both 2016 and 2018 (Magnusson Hanson et al. 2018). Registered responses for wave 2016 were 19,360 (47% of the cumulative cohort) and 17,841 (44% of the cumulative cohort) for 2018 (Magnusson Hanson et al. 2018). Out of these, 14,237 individuals responded to both 2016 and 2018 surveys between week 12 and week 22 resulting in a total of 28,474 observations after excluding participants with missing information on the outcome, i.e., symptoms of anxiety and covariates.

We restricted the sample to week 12 to 22 responses to ensure that the outcome reflected anxiety symptoms in the spring, while maintaining temporal proximity to the winter exposure period (November–February). Responses from June onwards (*n* = 714) were not considered, as they may have been influenced by contemporaneous weather conditions in the spring rather than by the preceding winter. This approach enhances comparability across survey waves and ensures alignment with our study aim of assessing springtime symptoms in relation to wintertime meteorological conditions.

### Solar radiation, precipitation and snow cover

Solar radiation, precipitation and snow depth measurements were obtained from the Swedish Meteorological and Hydrological Institute (SMHI). SMHI used the STRÅNG model (SMHIa 2025), a mesoscale model, with a horizontal resolution of 11 × 11 km and a temporal resolution of one hour, for assessing the amount of solar radiation. Solar radiation was measured based on incoming solar radiation on a horizontal plane. SMHI summed hourly values in watts per square meter (W/m²) to daily cumulative values in watt-hours per square meter (Wh/m²). For comparison with earlier studies, the solar radiation values were converted to megajoules per square meter (MJ/m^2^) by multiplying values with 0.0036. Precipitation data was measured as daily accumulated sums in millimeters (mm). Daily cumulative values were averaged over three (November to January) and four (November to February) winter months for each year for both solar radiation and precipitation. We chose to average the exposures over three-month and four-month periods because these represent the darkest months of the year in the Northern latitudes. This extended exposure window was also selected to capture the cumulative effects of weather patterns, which are more likely to influence mental health outcomes over longer periods rather than short-term fluctuations. As there are few studies focusing on winter-time exposures no definite exposure window has been defined. To account for potential differences between these definitions, both exposure windows were analyzed separately and served as complementary approaches. Snow depth is from SMHI-Gridclim datastet (SMHI Gridded Climatology) (SMHI 2025b). SMHI-Gridclim is a gridded climatological dataset covering the Nordic countries on a grid with 2.5 × 2.5 km horizontal resolution. The gridding was done using optimal interpolation of meteorological observations with statistically downscaled forecast fields as the first guess. Daily snow depth was measured in centimeters (cm). If a day had a snow depth of more than 0 cm it was coded as 1 and was summed as the number of days with snow over three months and four months. Snow was treated as such binary measure because the main focus was to examine the effects of presence of snow cover as a reflective surface that increases the amount of light, rather than the depth of the snow. After a snowfall, even a thin layer of snow reflects light substantially and brightens the environment similarly to a deeper layer. However, we acknowledge that this simplification does not capture potential differences in the intensity or duration of light reflection that might arise with greater snow depth.

No missing values were present in the exposure data, as both the STRÅNG reanalysis model and the SMHI-GridClim dataset provide spatially and temporally complete meteorological series. Exposure data were matched with SLOSH participants using residential municipality codes.

### Symptoms of anxiety

The self-reported outcome was assessed using SCL-ANX4, a subscale of the Symptom Checklist-25 (Holmgren et al. 2023; Søgaard And Bech 2009). Participants rated the extent to which they had experienced the following over the past week on a scale from 0 (not at all) to 4 (intensively): (1) nervousness or internal shakiness; (2) sudden feelings of fear without an apparent cause; (3) episodes of terror or panic; and (4) excessive worrying. Item scores were summed to create a total score ranging from 0 to 16. In line with recent population-based studies, including those using the SLOSH, a cutoff of ≥ 6 was applied to indicate clinically elevated anxiety symptoms (Blomqvist et al. 2024; Holmgren et al. 2023). In the original validation study, Christensen et al. 2005 demonstrated that a mean item score of ≥ 2.0 (equivalent to a sum score ≥ 8) identified clinically relevant anxiety disorders with a sensitivity of 82% and a specificity of 72% (Christensen et al. 2005). The use of a lower threshold (≥ 6) has since been widely adopted in epidemiological research to improve sensitivity to subclinical but functionally significant symptoms, while maintaining acceptable specificity. SCL-ANX4 has also shown strong psychometric properties, including scalability, monotonicity, construct validity, and predictive validity (Søgaard And Bech 2009). In our data, Cronbach’s alpha indicated good internal consistency for the SCL-ANX4 (2016 wave, α = 0.81; standardized α = 0.83; 2018 wave, α = 0.80; standardized α = 0.83).

### Covariates and possible effect modifiers

We identified factors previously recognized in the literature as potential confounders and effect modifiers in studies on meteorological variables and mental health outcomes (Kent et al. 2009; Kim et al. 2021; Raza et al., 2024; Son & Shin, 2021). Age and sex were used as covariates and obtained from registers. Age and sex were included because vulnerability to anxiety and other mental health symptoms is known to vary by age and sex, and these groups may also differ in their exposure and physiological sensitivity to weather-related changes (Cui et al. 2021; Kim et al. 2021; Yang et al. 2024).

Another covariate was region. Due to Sweden’s considerable geographical extent (1572 km from north to south), regional variations in weather conditions particularly, in solar radiation and snow cover, exist. To address this, we developed a region of residence variable using municipality codes, grouping municipalities into three broad categories (North, Middle, and South) based on their geographical location. For example, Kiruna belonged to the North and Malmö to the South category. This pragmatic classification was designed to reflect major climatic differences across the country rather than to follow strict latitude.

As possible effect modifiers we included civil status (married/cohabiting vs. not) and occupation that were self-reported. These reflect social support and material resources can buffer or exacerbate the effects of adverse weather on mental health, and socioeconomic position further influences housing, work conditions, and residential location, all of which affect exposure to meteorological factors (Kent et al. 2009; Umberson And Williams 1999). Statistics Sweden coded the occupations according to the Swedish Standard Classification of Occupations (SSYK 2012), which is aligned with the international ISCO-08 system. Using this classification participants were categorized into four occupational positions (low, intermediate, high, and self-employed) (Statistics Sweden 2012). The self-employed category represented only about 1% of the sample and was retained for consistency with the SSYK classification; however, its small size limits the statistical relevance of this group.

Job strain was another effect modifier. In the SLOSH surveys it was assessed using the Swedish Demand-Control Questionnaire, which includes five job demand items; (1) working at a fast pace, (2) working hard or intensively, (3) not having excessive workloads or needing too much effort, (4) having enough time, and (5) experiencing conflicting demands, and six job control items; (1) opportunities to learn new things, (2) use of a high skill level, (3) creativity or initiative, (4) repetitive tasks, (5) influence over what tasks to do, and (6) autonomy in how to perform them (Hanson et al. 2018). Job strain was defined as experiencing high demands (above the study-specific median for demand scores) combined with low control (below the study-specific median for control scores) (Sanne et al. 2005). The median-based definition is commonly used in SLOSH studies, as it ensures internal validity by accounting for the distribution of demands and control in the study population. However, comparability with studies that apply internationally standardized cut-off points (e.g., quartiles or external reference medians) may be limited, and prevalence estimates should therefore be interpreted with caution in cross-country comparisons. Job strain was used as an effect modifier as psychosocial stress at work is a well-documented determinant of mental health and may modify the health impact of weather-related exposures (e.g., lack of daylight during working hours) (An et al. 2016; Wang et al. 2023).

Further effect modifiers were health behaviors. In the SLOSH surveys, physical inactivity was assessed using the question: ‘How much do you exercise, including walking and cycling to and from work?’ Response options included: (1) never exercise, (2) move very little or take occasional walks, (3) exercise now and then, and (4) exercise regularly. Participants selecting the first or second option were classified as physically inactive; all others were considered physically active (Raza et al. 2021). While this binary classification is practical, it reduces nuance by collapsing variation in frequency and intensity of activity levels. Alcohol use was assessed in the survey using a modified version of the Cut-Annoyed-Guilty-Eye (CAGE) questionnaire (O’Brien 2008). The modified CAGE questionnaire included the following questions: (i) ‘Have you felt you should cut down on your drinking?‘, (ii) ‘Have people annoyed you by criticizing your drinking?‘, (iii) ‘Have you felt bad or guilty about your drinking?‘, and (iv) ‘Have you had a drink first thing in the morning to steady your nerves or alleviate a hangover?’ Respondents could answer ‘yes’ or ‘no’ to each question. Alcohol consumption was classified as risky if participants reported at least two problem drinking behaviors.

### Statistical analysis

We applied fixed-effects logistic regression to analyze the associations of solar radiation, precipitation and snow days during winter months in 2015–2016 and 2017–2018 with self-reported symptoms of moderate anxiety in spring 2016 and 2018 (defined as a score ≥ 6 on the SCL-ANX4 scale). This method treats each individual as their own control, thereby accounting for time-invariant characteristics such as sex, race, parental child-rearing practices, genetic makeup, and other factors through the design (Allison 2009).

We used crude (no covariates) model and model adjusted for age, sex, region, and the other meteorological variables that were not strongly correlated (*r* ≈ 0.5 or lower) with the one used as the exposure. Covariates were used as time-variant. Effect modification by all other variables were tested individually by adding one interaction term at a time to the adjusted fixed-effects logistic regression model (e.g., exposure × sex). An interaction term was included in the models for age, sex, civil status, job strain, occupational position, region, type of questionnaire (full-time vs. part-time), alcohol use, and physical activity. Effect estimates are reported as odds ratios (OR) with 95% confidence intervals (CI) for each unit increase in the exposure of interest. Unit for solar radiation is 1 MJ/m^2^, for precipitation 1 mm, and 10 days for the number of snow days. All analyses were conducted using SAS 9.4, employing PROC LOGISTIC for fixed-effects (conditional) logistic regression models.

## Results

In 2016, 9% of the participants reported clinically elevated symptoms of anxiety. The majority of the participants were women (58%), above 45 years of age (82%), had intermediate occupational position (48%), were gainfully employed (69%), and lived in southern Sweden (82%), reflecting a regional imbalance that was taken into account by including region as a covariate and testing it as a potential effect modifier. (Table 1). A total of 1,437 individuals experienced a change in their self-reported anxiety levels between the two waves. These changes were bidirectional, with some participants reporting higher and others lower levels of anxiety at follow-up, and were therefore treated as within-individual variation rather than a unidirectional trend.

**Table 1:** Characteristics of the study participants eligible for the analysis (*n* = 14,237) at their first measurement point

Variables	Frequency (percentage)
Outcomes	
Clinically elevated anxiety symptoms	1308 (9)
Other variables ^*^	
Women	8192 (58)
Region of residence	
North Sweden	874 (6)
Middle Sweden	1376 (12)
South Sweden	11,627 (82)
Age, years	
< 45	2521 (18)
≥ 45	11,716 (82)
Cohabiting	8560 (60)
Occupational position	
Low	3952 (29)
Intermediate	6578 (48)
High	3183 (23)
Self-employed	109 (1)
Job strain	2825 (20)
Type of questionnaire	
Employed less than 30% of full time, unemployed	4478 (32)
Employed more than 30% of full time	9795 (69)
Physically inactive	2516 (18)
Problem drinking	880 (6)
Region of residence	
North Sweden	874 (6)
Middle Sweden	1376 (12)
South Sweden	11,627 (82)

^*^ Sex, age, and region were included as confounders, while these and the other variables were also tested as potential effect modifiers

Descriptive statistics of the weather parameters are presented in Table 2, with mean values for all parameters being slightly higher in 2018. The low values for solar radiation reflect the very limited sunlight typical of Scandinavian winters at high latitudes. The correlation of solar radiation with precipitation was weakly positive (0.3), while with snow days it was highly negative (−0.7). The correlation between precipitation and snow days was − 0.1. To avoid collinearity, solar radiation and snow days were therefore not included in the same models.

**Table 2:** Descriptive statistics of weather parameters

Variables	2016			2018			
	Mean (SD)	Min	Max	Mean (SD)		Min	Max
Three-month daily average^a^							
Solar radiation (MJ/m^2^)	1.2 (0.3)	0.1	1.7		1.4 (0.4)	0.1	2.2
Precipitation (mm)	2.2 (1.1)	0.9	5.6		2.5 (0.7)	1.6	4.6
Number of snow days	41 (16)	21	92		48 (25)	2	92
Four-month daily average^b^							
Solar radiation (MJ/m^2^)	1.7 (0.3)	0.4	2.3		2.0 (0.5)	0.5	3.0
Precipitation (mm)	2.0 (1.0)	0.8	5.0		2.2 (0.6)	1.2	3.9
Number of snow days	61 (22)	15	121		74 (27)	15	120

^a^November-January

^b^November-February

### Associations between weather parameters and symptoms of anxiety

Odds ratios for three-month average solar radiation and symptoms of anxiety was below one, but the association was not statistically significant (OR 0.90, 95% CI 0.65–1.24). Similarly, exposure to three-month average precipitation was associated with an insignificant decrease in symptoms of anxiety (0.91, 0.79–1.05). We did not observe associations between snow days and anxiety symptoms (1.03, 0.95–1.10). Similar findings were observed between four-month average solar radiation, precipitation, and snow days (Table 3). Overall, these nonsignificant results suggest no clear evidence of associations in this sample, though they may also reflect limited statistical power to detect modest effects.

**Table 3:** Associations of solar radiation, precipitation, and snow days with clinically elevated anxiety symptoms, per unit increase in exposure

Exposure and covariates	Odds Ratio (95% Confidence Intervals)	
	Three-month average	Four-month average
Solar radiation		
No covariates	0.87 (0.63–1.21)	0.99 (0.77–1.30)
Precipitation, age, and region	0.90 (0.65–1.24)	0.94 (0.71–1.24)
Precipitation		
No covariates	0.90 (0.78–1.04)	0.87 (0.73–1.03)
Solar radiation, age, and region	0.91 (0.79–1.05)	0.87 (0.72–1.04)
Snow days		
No covariates	0.99 (0.93–1.05)	0.98 (0.92–1.03)
Precipitation, age, and region	1.03 (0.95–1.10)	1.00 (0.94–1.06)

We did not observe effect modification by sex, age, occupational position, civil status, job strain, physical activity, alcohol use, type of questionnaire, or region (all p-values for interactions > 0.05). No consistent non-significant trends were apparent across these analyses.

## Discussion

We investigated within-individual associations between solar radiation, precipitation (i.e., rain), and snow days during the winter months with self-reported anxiety in the following spring in Sweden, where winter months are predominantly characterized by cold and dark conditions. We observed an indication of protective association between higher exposure to solar radiation and precipitation on moderate anxiety, however, the confidence intervals were wide and included one. We did not observe association between snow days and anxiety. Individual, sociodemographic, and behavior-related health factors did not moderate the associations.

There are very few studies on the impacts of solar radiation on anxiety. A study from China observed a short-term (0–14-day) association between extremely low radiation and increased risk of outpatient visits due to anxiety (Zhang et al. 2023). Another study from Spain did not find significant correlations between annual mean solar radiation and anxiety episodes (Bulbena et al. 2005). These studies differ from ours in that they focused on short-term daily variation in clinical visits, whereas we applied a longitudinal within-individual design assessing cumulative winter exposures and self-reported symptoms in the following spring. Such methodological differences may partly explain the variation in findings. Our study findings do not prove that higher levels of solar radiation in winter months could have positive effects on self-reported anxiety symptoms though the effect estimate was in the protective direction. This aligns with our prior findings in this study population where higher solar radiation was protective of symptoms of depression (Raza et al., 2024). Thus, people might benefit from spending time outdoor when it is bright during winter months from November to February. One study has suggested that a whole-day lighting scheme that follows the natural light-dark cycle could be a solution to promote mood, at least among senior residents (Shishegar And Boubekri 2022). Mechanistic studies support such interventions, as the suggested underlying mechanisms for the associations between low levels of solar radiation and mental disorders include low serotonin and melatonin levels (Gałecki And Talarowska 2018), vitamin D deficiency (Geng et al. 2019), and disrupted cortisol-derived awakening response due to circadian rhythm disturbances (Germain & Kupfer, 2008; Jung et al. 2010). Furthermore, intervention studies have reported that exposure to natural light during the daytime synchronizes the internal circadian clock to solar time (Wright et al. 2013), preventing disruptions in circadian rhythms that may protect mental health. Another intervention study observed the lowest serotonin turnover by the brain during winter, with production rates directly associated with the prevailing duration of sunlight (Lambert et al. 2002).

We observed indications of a protective association between precipitation and symptoms of anxiety. This finding appears to be in contrast with the results concerning solar radiation, particularly if one considers there is less solar radiation during rainy conditions. However, our data showed only a weak positive correlation between precipitation and solar radiation, suggesting that light rain might occur with intermittent sunshine. Also, our precipitation variable might include melted snow, but we were unable to separate that from rain. Although there are not many studies on the relationship between precipitation and anxiety, the ones available reported inconsistent associations (Bulbena et al. 2005; Zhang et al. 2023). A Spanish study observed a protective effect of annual average of rain on emergencies attending a general hospital due to panic anxiety as the main complaint (Bulbena et al. 2005). A study from China, on the other hand, did not observe significant associations with short-term effects of extreme rain compared to no rain on outpatient visits due to anxiety, while the risk estimates were above one (Zhang et al. 2023). When comparing these results, one must consider differences in study designs, exposure periods, and study settings and locations, each characterized by distinct weather conditions and cultural perceptions. Our findings therefore underscore the importance of developing more refined precipitation measures that can better distinguish between rain and snow.

Our study is the first to investigate the association between snow days and any mental health outcome. Our findings did not indicate associations between snow days and symptoms of anxiety. This lack of association may be attributed to the limitations in our measurement of snow exposure. Future health research with more refined exposure metrics is essential, particularly in Scandinavian countries where snow is a predominant feature during the winter months. Snow may play a dual role with respect to mental health. On the one hand, snow cover increases the amount of available light through reflection, which could be beneficial in counteracting the darkness of Scandinavian winters. On the other hand, snow also enables outdoor activities such as skiing, walking, and other recreational pursuits that may promote social interaction, physical activity, and psychological well-being (Bielinis et al. 2019; Svensson et al. 2019). Conversely, heavy snowfall can sometimes limit mobility, restrict access to services, or increase stress related to commuting and daily routines. The balance between these potentially beneficial and adverse effects may help explain the absence of a clear association in our results. Future research with more refined exposure metrics, capturing both the intensity and consequences of snow cover, is therefore essential, particularly in Scandinavian countries where snow is a predominant feature during the winter months.

This study has several limitations. First, the outcome was self-reported in the spring, a few months after the exposure period. This time lag, combined with reliance on self-report, is a limitation of the study, as it may affect the validity of the outcome measure by introducing recall bias or misclassification of symptom timing. Although we have excluded the participants who responded after May, the timing between the exposure and outcome may not accurately reflect the true lag between wintertime weather exposure and the onset of anxiety symptoms. Second, 82% of participants were living in the southern Sweden which may have prevented us from seeing interaction with the region variable. Third, we used data on residential municipalities to link the weather data with the health data, which may have led to some exposure misclassification. While weather conditions are generally similar within municipalities, variation in factors such as altitude, coastal proximity, or local microclimates could still contribute to heterogeneity. We therefore cannot rule out that this spatial variability within municipalities diluted some associations. Fourth, despite controlling for some confounding variables and employing a fixed-effects design to account for time-invariant factors, such as genetics, we cannot exclude the possibility of unmeasured or residual confounding. Finally, our cohort primarily consists of a working population, making loss to follow-up unavoidable and potentially introducing a selection bias.

A key strength of our study was the use of repeated within-individual measurements to assess whether changes in exposure to meteorological variables during the winter months were linked to changes in anxiety symptoms. We also used a validated scale to assess symptoms of anxiety. Our study population was geographically representative of the working population in Sweden, and the survey data allowed us to account for potential confounding variables.

In conclusion, we observed no statistically significant associations between wintertime exposure to meteorological factors and self-reported symptoms of anxiety in the following spring. However, the effect estimates were to the protective direction for solar radiation and precipitation. While, to our knowledge, this is the first study to examine snow in relation to mental health outcomes, no association between snow days and anxiety was observed. Since our findings reflect rather specific Scandinavian context, further research from different geographical areas and populations is needed to understand whether wintertime weather conditions affect the level of anxiety.

## Data Availability

The datasets generated and/or analysed during the current study are not publicly available due to ethical issues. However, we agree to allow the journal to review our data if requested, given that such a request can be granted by Stockholm University based on relevant legislation at the time of the request. Corresponding author should be contacted for the data requests.

## Declarations

interests or personal relationships that could have appeared to influence the work reported in this paper.
